# Lessons for simulation-based education from social psychology

**DOI:** 10.1186/s41077-016-0007-0

**Published:** 2016-02-23

**Authors:** Ronnie J. Glavin

**Affiliations:** Scottish Centre for Simulation and Clinical Human Factors, Forth Valley Royal Hospital, Stirling Road, Larbert, FK5 4WR UK

**Keywords:** Social psychology, Educational theory, Reflection

## Abstract

Effective practice is informed by underlying theoretical models. Better awareness and understanding of such models can enhance reflection by practitioners on their current educational activities and so help drive the cycle of continuing improvement. In this article the author reflects on *three ways* in which a better understanding of social psychology gave insights into why some practices appeared to be more effective than others and some ways in which future practice could be altered. Social psychology places great emphasis on the importance of the situation in which people find themselves an how this impacts on their subsequent behaviour. The *three* areas specifically addressed in the article include factors which motivate and drive human activities, especially the importance of self-esteem. Secondly, the relevance of the fundamental attribution error, which looks at our tendency as humans to ascribe personal attributes as the cause of the behaviour of others rather than the influence of external events. The third area to be explored is the role of acquiring scripts or heuristics that can broaden the range of activities than can be performed at a subconscious or intuitive level. *For each concept, the author has included a brief illustration of its application to the practice of a simulation educator.*

**Electronic supplementary material:**

The online version of this article (doi:10.1186/s41077-016-0007-0) contains supplementary material, which is available to authorized users.

“Observations always involve theory” – Edwin Hubble

“Experience without theory is blind, but theory without experience is mere intellectual play” – Immanuel Kant

“Nothing is more practical that a good theory” – Kurt Lewin

## Introduction

In this article I would like to explore the ways in which some concepts from social psychology have helped me develop my roles as a teacher in both clinical and simulation-based education. 1

All of us who practice in simulation-based education of health care professionals utilise theories that guide and inform our behaviour, even if we are not always conscious of their nature or even their existence. The importance of such theories lies in their ability to provide a framework on which we can reflect, especially when our teaching has not gone well and we seek improvement for future practice. Of course, our theoretical models should be in a state of continual development and refinement, especially when we find that they do not adequately explain the phenomena that we observe in our educational practice.

I shall say a little about social psychology as a field of study and then explore the following three concepts: firstly, self esteem; secondly the Fundamental Attribution Error (FAE) and finally scripts and heuristics. However, I shall begin with a brief review of how I came into the world of simulation based education and some of the theories that I have employed.

## Background

I began my medical career in 1978 and throughout my anaesthetic training became increasingly interested in medical education. While working as a consultant anaesthetist I graduated as a Master of Philosophy in Educational Studies in 1993. My dissertation looked at the development of educational material that could help promote the values linked to patient safety within the UK anaesthetic training framework. In 1997 I was appointed as an Educational Co-Director to the Scottish Clinical Simulation Centre, which became active in early 1998. At this time the centre began a long and successful collaboration with Professor Flin of the Department of Industrial Psychology of the University of Aberdeen. My interest in psychology had been kindled during my M.Phil course and now, with more direct access to the world of industrial psychology I continued to read around psychology but in a relatively unstructured fashion.

A quotation from John Ruskin encapsulates my basic approach to the education of healthcare professionals [1] in general.

“Education is not about teaching people to know what they do not know:

It is teaching them to behave as they do not behave”.

I find this quote helpful because it places due emphasis on the role of values, key to the role of professionals in the workplace. My thesis supervisor expressed it in this way “Knowledge and skills give us abilities, but it is our value system that most influences when and how we choose to use those abilities.”

As educators we want to help learners choose to change how they behave and so some understanding of how we can encourage people to adopt some values while rejecting others is helpful. I used the methods described in David Krathwohl’s account of the Affective Domain of the Taxonomy of Educational Objectives [2]. Krathwohl describes a series of external and internal factors that influence the values which humans can adopt. The most basic factors are the potential impact of external reward or punishment. This is followed by a desire to conform to the group of whose membership we aspire. The third stage is where we accept the value for its intrinsic benefit and the fourth stage relates to how the particular value being promoted fits into the hierarchy of all of the values that any one individual holds. I found that I could make more sense of this scheme by considering the motivators or drivers that have an impact on human behaviour; upon which I shall now elaborate.

## Motivators in education

A very simple linear narrative of psychology and motives could begin with Freud thinking about how various subconscious desires influenced our behaviours, and how our brains kept these in some sort of balance to allow humans to function in co-operative society; the relative roles of the id, the ego and the superego. These motives or drives were thought of as internal [3]. The next group to formally study drives were the Behaviourists [3], who wanted to take a more objective, positivistic approach to the response of organisms to stimuli. Luminaries of this group, such as B.F. Skinner, viewed the brain as a ‘black box’ that could not be examined directly. However, by presenting animals with stimuli, positive or negative, one could study such factors as the strength or duration or frequency of stimuli on the response of the test subjects. That response could be measured in terms of how quickly it was achieved, how quickly it dissipated and the impact of subsequent reinforcement of the initial stimulus. The behaviourists relied on some internal driver, such as hunger, to provide a motive for their subjects: hungry rats would be encouraged to find a route through a maze to locate a piece of food. They did not deny the existence of internal motives, the held the view that proper scientific study of the workings of the ‘black box’ was not possible at that time.

**Fig. 1:**
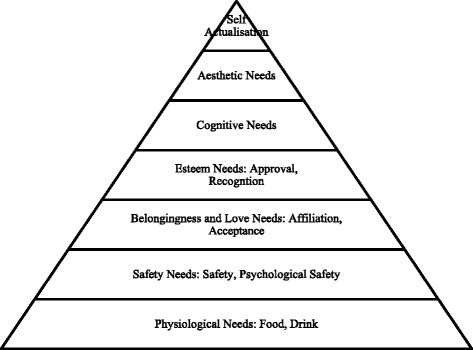
Maslow’s Needs Hierarchy [3]

The next psychologist I wish to consider is Abraham Maslow [3]. Most of us who have undergone formal educational courses are familiar with Maslow’s need hierarchy (Fig 1) [3]. Maslow did not conduct the research required to validate the hierarchy and although others, such as Deci [4], have done more work on this field I find that Maslow’s hierarchy has provided a model that has helped me make sense of professional development. The top level – self actualisation – surely fits in with the notion that professionals have about themselves. Their sense of who they are, including their sense of worth, is linked to their professional role. I can think of many doctors and nurses who have relinquished meal breaks (bottom level) because of urgent clinical demands. Level 4 – approval and recognition – is consistent with the notion of learners seeking to join a community of professionals. I also believed that professionals use their experience to seek to improve their performance and in this I was influenced by the writings of Jarvis [5], Knowles [6] and Kolb [7]. I also believe that reflection is facilitated when practitioners share a common vocabulary that applies to the area under study. If discussing novels with a friend I may refer to characterisation, plot, dialogue, mood and so on. In 2012 I undertook formal teaching in Social Psychology.

## Social psychology – what is it?

A simple definition of psychology is ‘the scientific study of behaviour and the mind’ the term ‘mind’ can be substituted by the term ‘mental processes’ [3]. Social psychology is defined as ‘the scientific investigation of how the thoughts, feelings and behaviours of individuals are influenced by the actual, imagined or implied presence of others’ [8]. I would like to illustrate this definition by describing a famous experiment – The Good Samaritan Study [9]. In this study students at a New England seminary were allocated into two groups. Group A were asked to give a short talk on life in a seminary, while those in Group B were asked to give an account of the parable of the Good Samaritan. Half of each group were informed that they were running late and should hurry over to the lecture theatre; the other half was informed that they had some time but should go over early to ensure that everything was prepared. During the walk to the lecture theatre each student encountered a confederate of the investigators simulating a medical emergency. The investigators measured a number of variables for each group but found that the factor that had most impact on the behaviour of the students in terms of whether they would offer assistance or not was the perception of being early or late. Only ten per cent of the ‘late’ group offered assistance whereas over sixty per cent of the ‘early’ group offered assistance. This study, and others of a similar nature suggest that the situation a person perceives them self to be in has a much greater impact on their behaviour than other predisposing factors, such as knowledge, personality attributes etc. Social psychology studies how external factors can interact with an individual’s internal drivers. I would like to explore three concepts in further detail: self esteem, the fundamental attribution error and scripts and heuristics. *I have chosen these three because they had the biggest impact on changes that I made to my own practice and because they helped me to explain some of my actions to other simulation centre faculty members*. I shall begin with self esteem.

## Self-esteem

Self esteem can be thought of as our sense of self worth and in the educational realm can be thought of in terms of a ‘need for competence’ [4]. One could speculate that the seminary students wanted to be regarded positively; *for those in the ‘late’ group the thought of being even later for the lecture could clash with that sense of positive regard. Maslow’s hierarchy refers to self-esteem in level 4 but I argue that in the context of professionals their identification of self with the professional role is also consistent with self-actualisation. Indeed, it is some of the limitations of my use of Maslow’s hierarchy in my practice that encouraged me to adopt other theoretical models. In the early days of simulation based education experiences a recurring scenario played out. Individuals holding important educational posts in Scottish Anaesthesia would ask if they could attend and observe. This was always accompanied by the phrase “you won’t make me do a scenario, will you?” I asked why and came to realise that the threat to their sense of professional status was so great that they were not prepared to put it to the test*. Experienced professionals who do undertake scenario based education may change their behaviour to minimise this risk to their need for competence. They may play the game of ‘spotting’ the scenario, they may blame external factors; “It didn’t look real”, “It didn’t behave the way it should have”. We have all heard these comments, especially if things have not worked out so well during the scenario. So this is a very real concept and one of the approaches I found helpful came from reading Carol Dweck’s work on positive psychology [10]. Dweck describes a study in which primary school age children were given a problem in mathematics to solve. Some of the group were told that they were very good at mathematics and had above average mathematical ability. Other members of the group were told that they were very hard working and had above average levels of persistence. When the group were presented with further problems those in the ‘above average mathematical ability’ group were more reluctant to tackle them than those from the ‘above average persistence’ group. Dweck argued that the ‘above average mathematical group’ had more to lose because if they didn’t solve the problems then their self esteem as better mathematicians would be challenged. The more persistent group had nothing to lose because failure to solve the problem would not negate their self-esteem. Dweck refers to the ‘above average at maths’ mindset as a fixed mindset in contrast to the ‘above average at persistence’ mindset as a growth mindset. What Dweck did was to reframe the mindset from a fixed one to a growth one and I found this concept very helpful when dealing with experienced professionals in scenario based simulation. I explored some notions of professionalism with the group during the introduction session and from the discussion made the explicit statement that professionalism includes the desire to improve one’s professional performances (consistent with self-actualisation) and this means being able to learn from one’s performance. The focus of the course moves from concentrating on individual performance, without ignoring that component, to thinking about strategies that may work in future clinical encounters. This is explored further in the Vignette in Additional file 1.

I am old enough to have experienced teaching by humiliation as a medical student and when I reflect upon the strategies that I and my colleagues adopted to avoid the threats to our self-esteem I can only think that such behaviours (say nothing, make up facts, not turn up) were not ones that would promote a good educational environment. Self-esteem deals with how the individual perceives his or her standing or competence. The next area looks at how others may judge an individual. This takes us onto the Fundamental Attribution Error.

## Fundamental Attribution Error

The next area I want to explore is the Fundamental Attribution Error [8] (FAE). I shall illustrate with a fictitious example. Let us imagine that Person A is spending the first day in a new healthcare job. This job is similar to one previously held by that person in a different location. At a break Person A’s new colleagues ask the individual to join them for lunch. After a few minutes Person A does join the others. During the meal Person A neither joins readily in conversation nor appears to following those topics of conversation discussed by the others. Person A leaves the lunch table ten minutes before the others without comment. The new colleagues agree that Person A appears to be aloof and unfriendly, almost to the point of being antisocial. However, another colleague who had met Person A previously, expresses surprise and states that such behaviour was not typical from previous encounters. Indeed, Person A was lively, attentive and very popular with colleagues. How might we explain this discrepancy? This colleague talks to Person A and discovers that Person A was up most of the night with a sick child, who required hospital admission but is now in a stable condition. Person A chose to come to work because it was the first day and Person A’s spouse could be present in hospital with the child. On the way to work Person A was also involved in a minor road traffic accident, resulting in no personal harm but a future garage repair bill is likely. So which is the real Person A? Is person A the quiet, aloof, retiring individual or the lively, friendly and attentive individual? What is different? Well the circumstances are different and when we learn of Person A’s predicament we are much more likely to be understanding of Person A’s behaviour at lunch time. So the FAE consists of attributing behaviour to personal predispositions, such as personality factors, rather than attributing the circumstances in which an individual finds them self. As we have seen previously social psychology suggests that the circumstances, the situation, has a much more important bearing on behaviour than the personal characteristics of the individual.

*How does this the FAE fit in with my notion of motives? My working model is that we have drivers, such as the need to preserve self-esteem. Social psychologists argue that some aspects of the social situation will activate some of these drivers. However, they operate at a level that is normally inaccessible to our conscious thought processes. By way of contrast we are aware of differences in people and easily (if not always accurately) ascribe personality types to individuals we do not know well, even if we have barely met the person.* Evolutionary psychologists [11] hypothesise that when our human ancestors encountered strangers they had to quickly decide whether they were hostile or not and failure to identify hostile individuals could have negative consequences for that individual’s ability to contribute to the gene pool.

For part of my professional life I was responsible for the development and running of courses for doctors in Scotland who had to carry out clinical and educational supervision roles. I was impressed by how often the FAE came up and how readily senior clinicians attributed behaviours of their trainees to personal failings *- “that doctor is lazy”; “that doctor is a troublemaker”; “that doctor is not very bright” and so on.* So what can we do about the FAE in simulation based education? The first and most important point is to be aware of it. As simulation centre faculty will be judging the participants on our courses on first acquaintance; that is what we do as humans. What we must not do is ascribe their behaviours during the scenario to personality or cultural factors without exploring the impact of their perceptions of what was happening in the scenario. Behaviour is more likely to be due to the circumstances occurring during the course and the scenario than due to personal or cultural characteristics. In other words, the situation is more likely to elicit a response from deeper drivers than from more superficial influences such as personality characteristics. If we as facilitators think that the behaviour of a candidate was strange or abnormal then rather than label them instantly as having a ‘defective personality combination’ we should attempt to find out more about how that person perceived the circumstances. This approach is consistent with the Advocacy Inquiry method [12]. This is explored further in the Vignette in the Additional file 1. This instant judgement applies equally to our assessment in the work place of trainees that we do not know well. *The notion of making quick judgements doesn’t just apply to the personalities of other people. It applies to many aspects of life, especially professional life and this brings me onto the third and final concept of this review – the use of scripts and heuristics.*

## Scripts and Heuristics

The final concept I mentioned was the use of Scripts and Heuristics. This is a very big area in medical education just now and follows on the work of Kahneman and Twersky [13]. Heuristics are rules of thumb that we have developed which allow us make better use of our subconscious mind – described as fast thinking. I think of scripts as a subset of heuristics. The key features of a script are firstly that actors know what they are supposed to do and say and secondly that there are cues letting the actors know when they are expected to respond or react. Social Psychologists often use the restaurant script as an example. A typical example may go like this.

Customer – “we have a table booked for 7 pm under the name of Smith”

Restaurant staff – having checked bookings list “Come this way, here are the menus, the waiter will tell you about the specials”

Front of House Staff – “Can I get you something to drink”

Customer – “Can I see the wine list?”

Etc.

*The above script may win no prizes for literary merit but it contains those two concepts of firstly, knowing what to expect from the occasion and secondly, how to respond to the actions of the restaurant staff.* Two other driving forces – minimising ambiguity and reducing cognitive work load [14] come into play here. We reduce cognitive load by making the process automatic; that is, the pattern and the specifics of the script are transferred from our conscious working memory to our long term memory and can be recalled when appropriate. We reduce ambiguity by remembering how the sequence is supposed to play out. Of course, there are different kinds of restaurants with different patterns of expected behaviour – buffet, self service etc. and so we build up a repertoire of scripts that we can use for these different circumstances and cues will determine which script we call upon to use. Formica tables, plastic tables and cutlery and several queues at a serving counter will evoke one script, smart furniture, linen napery and the presence of a sommelier will evoke an entirely different script but they are still part of the set of restaurant scripts.

*I find this model, in which a person builds up a repertoire of scripts related to professional encounters, very helpful because it expands on the Novice to Expert model described by the Dreyfus Brothers* [15]*. The Novice to Expert model describes changes that take place in the cognitive processes as a professional moves from being a novice (relying heavily on rules) to becoming an expert (making extensive use of cognition). The relevance of this model to healthcare was described by Benner* [16]*. Interestingly, Social Psychologists argue that the ‘fast thinking’ associated with scripts and heuristics is also connected with our willingness as humans to ascribe stereotypes to other people and this may be a contributing factor to the Fundamental Attribution Error.*

We can experience something similar in a clinical setting. Let us imagine a medical student with no personal experience of asthma learning the management of someone suffering an acute asthma attack. The student will probably learn guidelines as a basic script but the more patients the student meets and the greater their involvement in the management then the richer the repertoire of scripts for managing a patient with asthma will become. At the most basic level the student learns an algorithm, which can be thought of as set of rules, and like all sets of rules are helpful to learners by reducing ambiguity. However the guidelines only provide one version of a script and it is only through clinical experience that the scripts become richer and the repertoire of scripts builds up. Some interactions will be common to the majority of these scripts – administer high inspired concentration oxygen, give bronchodilators and so on. Different types of clinicians will have acquired different ranges of scripts for the management of patients with acute asthma – family doctors will acquire a lot of experience of managing patients with asthma and their families and carers but may not see so many severe acute attacks; whereas, intensivists will have a lot of experience of patients with very severe attacks of asthma but much less experience of mild attacks.

This model – the development of scripts – can help us in our design of scenarios in simulation based education. At the level of the novice, where rules are dictating the interactions in a very basic script, strong cues may be helpful. If our wish as educators is to help the learners establish a basic script in long term memory then knowing when to intervene may be helpful. Certain models of simulator have features such as LEDs that are intended to represent the blue of cyanotic peripheries or the red dots of an allergic rash. I am conscious that in my own centre we have often exaggerated physiological values to act as cues to bring out a response from the participants. We have made the heart rate is a bit faster than it probably would be, the blood pressure is a bit lower, SpO2 is a bit lower and so on. I have always held concerns that we may be promoting a behaviourist model of conditioning. I think it is less important if learners are exposed to such experiences infrequently but if we wish to reinforce the place of such algorithms in long term memory and choose to delineate the intervention points, the points at which the learner is expected to initiate an action, we may reinforce an inappropriate pattern.

Another model that may help explain my concern is that of signal to noise ratio. What we are attempting to do in our scenarios is to make the signal so loud so that it stands out above the background ‘noise’ and so becomes less ambiguous. This may be acceptable for novices who are learning a script that is based on rules. However, when we are delivering scenario based courses for more experienced health care professionals then the scripts that we seek to create in our simulated environment may not be faithful to the repertoire of scripts residing in the long term memories of our participants. Such learners are likely to have acquired the ability to discern more subtle signals from the noise. In some cases I suspect that the cues that would activate a particular script in real life may not be able to be recreated in the simulated environment. In some cases this may be down to limitations of the hardware or *even the simulated actors, simulated patients or confederates in the scenario*. These issues by themselves are not new but maybe we have to add ‘script fidelity’ to our ever burgeoning dimensions of fidelity as yet another factor to consider when developing courses. As a former obstetric anaesthetist there would be subtle signs but important cues from women undergoing caesarean section under regional anaesthesia that a manikin or even a simulated patient would struggle to replicate. I have no simple solutions for this challenge although I have used the limitations of the manikin and simulated environment as a way of setting an agenda for discussion in courses with experienced clinicians. By asking a group what they would expect to observe, and when they would intervene one can help these clinicians explore their own scripts and so reflect upon them.

Our scripts are unique to us because they are built from our own experiences. I think that one of the ways of learning from others is to make aspects of their scripts more explicit and I think that one of the strengths of scenario based simulation is to use the scenario as a way of bringing scripts from long term memory into the working space of short term memory. I wrote earlier that this also has the advantage of moving the focus away from that of the performance of the individual learner in a scenario and putting the focus on the discussion generated from the performance. This helps with the self-esteem of the learner but the script / heuristic model also helps me reflect on why some discussions went particularly well and other did not. The use of the script / heuristic model may help the facilitator concentrate on some of the more salient components of scripts, such as the way in which clinicians anticipate that the course of an event will follow and how and when they would intervene, update their model and so in. I find this especially interesting because it links this model with the cognitive non-technical skills of situation awareness and decision making. I think that simulation-based education can help with continuing professional development and maintenance of competence by helping healthcare professionals learn aspects of practical management from their peers as well as helping individual practitioners reflect upon their own strengths and weaknesses.

I explore this further in the Vignette in Additional file 1.

## Summary

The person and the situation summarises the main thrust of what social psychology is all about. We create situations when we create scenarios in our simulation roles. As health care professionals we have considerable ability and opportunity to influence the behaviours of our learners and to help them learn by facilitated reflection of such behaviours. As humans our behaviours are complex because not only do we each vary in sensitivity to those factors that may provoke a pattern of behaviour but the very patterns themselves will be influenced by factors such as cultural conditioning and personality dispositions. This is not intended to be a comprehensive review of social psychology but I hope that I have shown ways in which my own practice has been influenced by my interpretations of the material I studied. I believe that the greater our understanding of these factors then the more useful our own models and theories will become in helping us develop our role as educators and as health care professionals.

## Additional file

Additional file 1: **Vignette.** (DOCX 14 kb)

## Abbreviations

ANTS: Anaesthetists’ non-technical skills
FAE: Fundamental Attribution Error
SpO2: Peripherally measured oxygen saturation

## Footnotes

1 The Editor-in-Chief and Senior Editors commissioned this paper because of his expertise in healthcare simulation education and his recent formal studies in social psychology. As Dr Glavin notes, educational practices are underpinned by theories but these are often not made explicit in courses about simulation education. We believe this combination of simulation expertise and recent exploration of social psychology enables identification of concepts relevant for simulation educators.
