# Supplementary material for: Lessons for simulation-based education from social psychology
Source: Adv Simul (Lond). 2016 Feb 23;1:7. doi: 10.1186/s41077-016-0007-0 (PMC5806458; doi:10.1186/s41077-016-0007-0)
Supplement: Supplementary file 1 — Vignette. (DOCX 14 kb) [file 41077_2016_7_MOESM1_ESM.docx]

LESSONS FOR SIMULATION-BASED EDUCATION FROM SOCIAL PSYCHOLOGY

ADDITIONAL FILE 1

ILLUSTRATIVE VIGNETTE OF THE APPLICATIONS OF THE THREE CONCEPTS TO SIMULATION BASED EDUCATION

CONTEXT

The setting is one day course for career grade anaesthetists or trainees in final two years. The educational aims of the course are promotion of the use of the Anaesthetists’ Non-Technical Skills (ANTS) Framework. Personnel consist of six participants, two faculty members and technical support.

COURSE INTRODUCTION

I include the following points during the general introduction:

“You have chosen to attend this course and that suggests to me that you take your own professional development seriously. I begin on the basis that you are already effective practitioners who want to become even more effective in the area of Non-technical skills. Each of you will be lead consultant in one of the scenarios. The scenarios will provide some concrete material that will not only help you reflect on your individual performance but also help us exchange strategies and actions that have proved useful previously. The scenarios are intended to be challenging because by leaving your comfort zone you are more likely to learn something about your own resources.”

Commentary

The intention is to minimise the threat to self-esteem by acknowledging some of the existing professional strengths of the participants and to avoid over concentrating on the performance of the individual. This does not mean that aspects of individual performance are not reviewed but the main educational objective is to share their experiences during a facilitated discussion and to attempt to create an environment in which peers are sharing practice. Note, this would not apply to a course aimed at trainees in the early stage of training, where the educational goals would differ.

SCENARIO 1 - BACKGROUND

During this scenario the hot-seat participant is informed that he/she is the on-call consultant anaesthetist working in an office in the hospital in the evening midweek. The telephone rings; the trainee anaesthetist on call (played by faculty member) is requesting assistance in the management of a patient undergoing emergency laparotomy for a ruptured large bowel diverticulum and the trainee suspects that the patient is becoming more septicaemic. The learning outcomes focus on those elements of the ANTS framework in the categories of Task Management and Team Working. Let us imagine that the hot seat consultant, after a short discussion with the trainee, decided to insert central venous canulation and delegate perioperative care of the patient to the trainee while the procedure was being performed.

Commentary

Social psychologists conduct experiments by manipulating situations and observing the effects upon the subjects of those experiments. Writing and directing a scenario for simulation-based education shares many of those features. In this case the scenario is designed to challenge the ability of the participant to manage the task of providing per-operative care for a patient whose clinical situation is changing. The educational value comes from helping the participant explore the behaviours adopted in response to the situation created by the scenario designer and director. If the scenario was designed to look at behaviours relating to Situation Awareness then some aspects of the situation would be changed. For example, we may have the patient on medication that would prevent the fast heart rate response. Different individuals will behave differently but the educational goals will be best served by helping that participant explore which aspects of the situation as perceived by the participant brought about the behaviours that were observed. Awareness of the Fundamental Attribution Error is my safeguard from lazily ascribing behaviour to some personal characteristic of the participant.

SCENARIO 1 – DEBRIEFING

I would expect a consultant anaesthetist to delegate the task of cannulation to a trainee with some experience because it is a procedure commonly performed and it would allow me as the consultant and team leader of the anaesthetic management component to monitor the whole situation and intervene as necessary. As facilitator I want to find out why this behaviour was chosen. I would begin by asking if that action was consistent with the participant’s behaviour in the real clinical world. If so, then I would ask the other participants to say what they normally do in this situation. This can help ‘normalise’ the behaviour, which is an underrated feature of such courses. I would then explore in more detail why this was chosen, why this course of action made sense to the participant. I would also ask the other participants for some examples of when they would delegate and when they wouldn’t.

The major challenge offered by this scenario is that a lot of tasks require to be performed and the ability of the participant to prioritise, delegate, buy time, collaborate with the surgical team, involve other interested parties etc. As facilitator I would use the experience of the hot seat participant to activate the scripts that others have acquired during their management of such cases. In addition to the scripts I would also invite the other participants to describe which behaviours were effective in the above. These would be further discussed when coming up the take home messages of the hot-seat participant for this scenario and those chosen by the other participants.

Commentary

The above needs time and when designing such a course I would tend to go for short scenarios – ten to fifteen minutes will allow the participant to become immersed but provide sufficient material to provoke discussion as described above. Our experiences differ and so our scripts and working practices differ. Other factors, including the size and layout of the hospital will influence the resources available to participants. An important educational aim in such courses is to provide participants with some further options to explore when prevented with future clinical challenges. The underlying science of the medical condition can be explored in other settings, departmental meetings, grand rounds etc; but the above type of course allows discussion on the ‘art’ of the profession and I see that art in terms of developing a rich repertoire of scripts.
